# Promoter Architecture Differences among *Alphaproteobacteria* and Other Bacterial Taxa

**DOI:** 10.1128/mSystems.00526-21

**Published:** 2021-07-13

**Authors:** Kevin S. Myers, Daniel R. Noguera, Timothy J. Donohue

**Affiliations:** a Wisconsin Energy Institute and Great Lakes Bioenergy Research Center, University of Wisconsin—Madison, Madison, Wisconsin, USA; b Department of Civil & Environmental Engineering, University of Wisconsin—Madison, Madison, Wisconsin, USA; c Department of Bacteriology, University of Wisconsin—Madison, Madison, Wisconsin, USA; KU Leuven

**Keywords:** bioinformatics, motif prediction, promoters, transcription

## Abstract

Much of our knowledge of bacterial transcription initiation has been derived from studying the promoters of Escherichia coli and Bacillus subtilis. Given the expansive diversity across the bacterial phylogeny, it is unclear how much of this knowledge can be applied to other organisms. Here, we report on bioinformatic analyses of promoter sequences of the primary σ factor (σ^70^) by leveraging publicly available transcription start site (TSS) sequencing data sets for nine bacterial species spanning five phyla. This analysis identifies previously unreported differences in the −35 and −10 elements of σ^70^-dependent promoters in several groups of bacteria. We found that *Actinobacteria* and *Betaproteobacteria* σ^70^-dependent promoters lack the TTG triad in their −35 element, which is predicted to be conserved across the bacterial phyla. In addition, the majority of the *Alphaproteobacteria* σ^70^-dependent promoters analyzed lacked the thymine at position −7 that is highly conserved in other phyla. Bioinformatic examination of the *Alphaproteobacteria* σ^70^-dependent promoters identifies a significant overrepresentation of essential genes and ones encoding proteins with common cellular functions downstream of promoters containing an A, C, or G at position −7. We propose that transcription of many σ^70^-dependent promoters in *Alphaproteobacteria* depends on the transcription factor CarD, which is an essential protein in several members of this phylum. Our analysis expands the knowledge of promoter architecture across the bacterial phylogeny and provides new information that can be used to engineer bacteria for use in medical, environmental, agricultural, and biotechnological processes.

**IMPORTANCE** Transcription of DNA to RNA by RNA polymerase is essential for cells to grow, develop, and respond to stress. Understanding the process and control of transcription is important for health, disease, the environment, and biotechnology. Decades of research on a few bacteria have identified promoter DNA sequences that are recognized by the σ subunit of RNA polymerase. We used bioinformatic analyses to reveal previously unreported differences in promoter DNA sequences across the bacterial phylogeny. We found that many *Actinobacteria* and *Betaproteobacteria* promoters lack a sequence in their −35 DNA recognition element that was previously assumed to be conserved and that *Alphaproteobacteria* lack a thymine residue at position −7, also previously assumed to be conserved. Our work reports important new information about bacterial transcription, illustrates the benefits of studying bacteria across the phylogenetic tree, and proposes new lines of future investigation.

## INTRODUCTION

The transcription of DNA to RNA is vital for cells to grow, develop, and respond to environmental stimuli. In addition, transcription can be harnessed to engineer microbial strains that sequester carbon dioxide or nitrogen gas or produce fuels, chemicals, pharmaceuticals, and materials (1, 2). Despite the central role of transcription, most of our understanding of this process in bacteria has come from analysis of a few species. We are interested in understanding transcription across bacteria, especially those with activities of agricultural, environmental, medical, and industrial importance.

Transcription depends on RNA polymerase (RNAP) binding in a site-specific manner to promoter DNA sequences, and it can be regulated by protein factors and small-molecule ligands (3, 4). Biochemical, structural, and genetic studies have shown that bacterial RNAP is typically composed of core subunits (β, β′, ω, and α_2_) and a specificity subunit (σ) that allows a holoenzyme to recognize promoter DNA sequences (5, 6). Bacteria often contain multiple σ factors, each directing a different RNAP holoenzyme to recognize specific DNA sequences in promoters (7–10), thereby allowing individual RNAP holoenzymes to transcribe different gene sets (11–16). It is also well known that differences in promoter sequences can influence either RNAP holoenzyme binding or subsequent steps in transcription initiation (7–9, 17–20).

While bacteria often contain multiple σ factors (21, 22), a housekeeping σ factor (often called σ^70^ in reference to the molecular weight of the Escherichia coli protein) is known or predicted to recognize promoters containing a −35 and a −10 motif in bacteria (numbers indicate the number of bases upstream of the transcription start site [TSS]) (21–27). Housekeeping σ factors are typically essential proteins because of the number and diversity of genes that require σ^70^ for transcription (25). In E. coli, highly conserved nucleotides in the −35 (^−35^TTG^−33^) and -10 (^−12^TATAAT^−7^) elements of the promoter carry out base-specific interactions with amino acids in the σ^70^ subunit (5, 22), suggesting that these or similar protein-DNA contacts are important in the process of transcription across the bacterial phylogeny (25).

Despite this, there is emerging evidence that some aspects of the paradigm for σ^70^-dependent transcription developed in the gammaproteobacterium E. coli may not apply across the bacterial phylogeny. For example, in several groups of bacteria, maximal transcription from σ^70^-dependent ribosomal (rRNA) operons *in vitro* requires CarD, an RNAP-binding protein that interacts with promoter DNA just upstream of the −10 hexamer (28–31). CarD family members are found in several bacterial phyla or classes, including *Actinomycetes*, *Aquificae*, *Cyanobacteria*, *Deinococcus*-*Thermus*, *Firmicutes*, *Spirochaetes*, and *Thermodesulfobacteria*, as well as *Alphaproteobacteria* and Deltaproteobacteria (30, 32). When tested, *carD* is often an essential gene and its loss or depletion reduces expression of many genes, supporting a hypothesis that CarD has a role in controlling expression of numerous cellular processes and pathways (33–36).

Indeed, it was recently shown that promoters for the σ^70^-dependent rRNA operons and other genes from the alphaproteobacterium Rhodobacter sphaeroides were activated by CarD*_Rsp_ in vitro* (96). In addition, the requirement for CarD for maximal transcription was found to be due, in part, to the absence of a thymine at the last position of the −10 motif in these promoters (96). Further, analyses of published genome-scale transcription start site (TSS) data sets suggest differences in the −10 motif recognized by the housekeeping σ factor between R. sphaeroides, a few other *Alphaproteobacteria*, and a limited number of well-studied bacteria (37, 96). In this work, we leveraged published genome-wide TSS data from organisms across the bacterial phylogeny to predict the sequences of −35 and −10 elements of their σ^70^-dependent promoters (37–44). We also used the lack of a thymine at position −7 to predict the cellular functions encoded by genes that are known or predicted to require CarD to activate transcription across the bacterial phyla. Based on this, we propose that differences in promoter architecture and the presence of CarD play a previously unrecognized role in transcription of essential and other genes in *Alphaproteobacteria* and possibly other bacterial phyla.

## RESULTS

### TSS-based bioinformatic predictions of promoter sequences across the phylogeny.

Past genome-scale predictions of bacterial promoter sequences have often been made without knowing the precise site of transcription initiation by RNA polymerase that is provided by transcription start site (TSS) data sets. High-throughput TSS sequencing (TSS-seq) has recently been used to provide genome-scale coordinates for the initiation sites for bacterial transcription units at the nucleotide level (37, 38). We gathered publicly available genome-scale TSS-seq data sets from a variety of bacterial species in order to predict bacterial promoter sequences and to ask if they were similar across the phylogeny. The published bacterial TSS-seq data sets that we analyzed included several thousand experimentally determined TSSs from *Actinobacteria* (40, 41), *Alphaproteobacteria* (37, 38, 43), *Betaproteobacteria* (39), *Firmicutes* (44), and *Gammaproteobacteria* (42) (Table 1).

**TABLE 1:** Summary of TSS data analyzed and annotated σ factors for organisms studied

Species	Phylum or class	No. of:			Genome accession no.
		TSS analyzed	TSS conditions	Annotated σ factors	
Mycobacterium smegmatis	*Actinobacteria*	2,139	3	26	NC_008596.1
Streptomyces coelicolor	*Actinobacteria*	3,570	44	63	NC_003888.3
Caulobacter crescentus	*Alphaproteobacteria*	2,726	8	16	NC_011916.1
Novosphingobium aromaticivorans	*Alphaproteobacteria*	2,301	2	12	NC_007794.1
Rhodobacter sphaeroides	*Alphaproteobacteria*	3,015	2	17	NC_007493.2
Zymomonas mobilis	*Alphaproteobacteria*	3,940	3	5	NZ_CP023715.1
Burkholderia cenocepacia	*Betaproteobacteria*	6,598	1	20	AM747720.1
Bacillus subtilis	*Firmicutes*	5,601	1	18	NC_000964.3
Escherichia coli	*Gammaproteobacteria*	2,702	3	7	NC_000913.3

**FIG 1:**
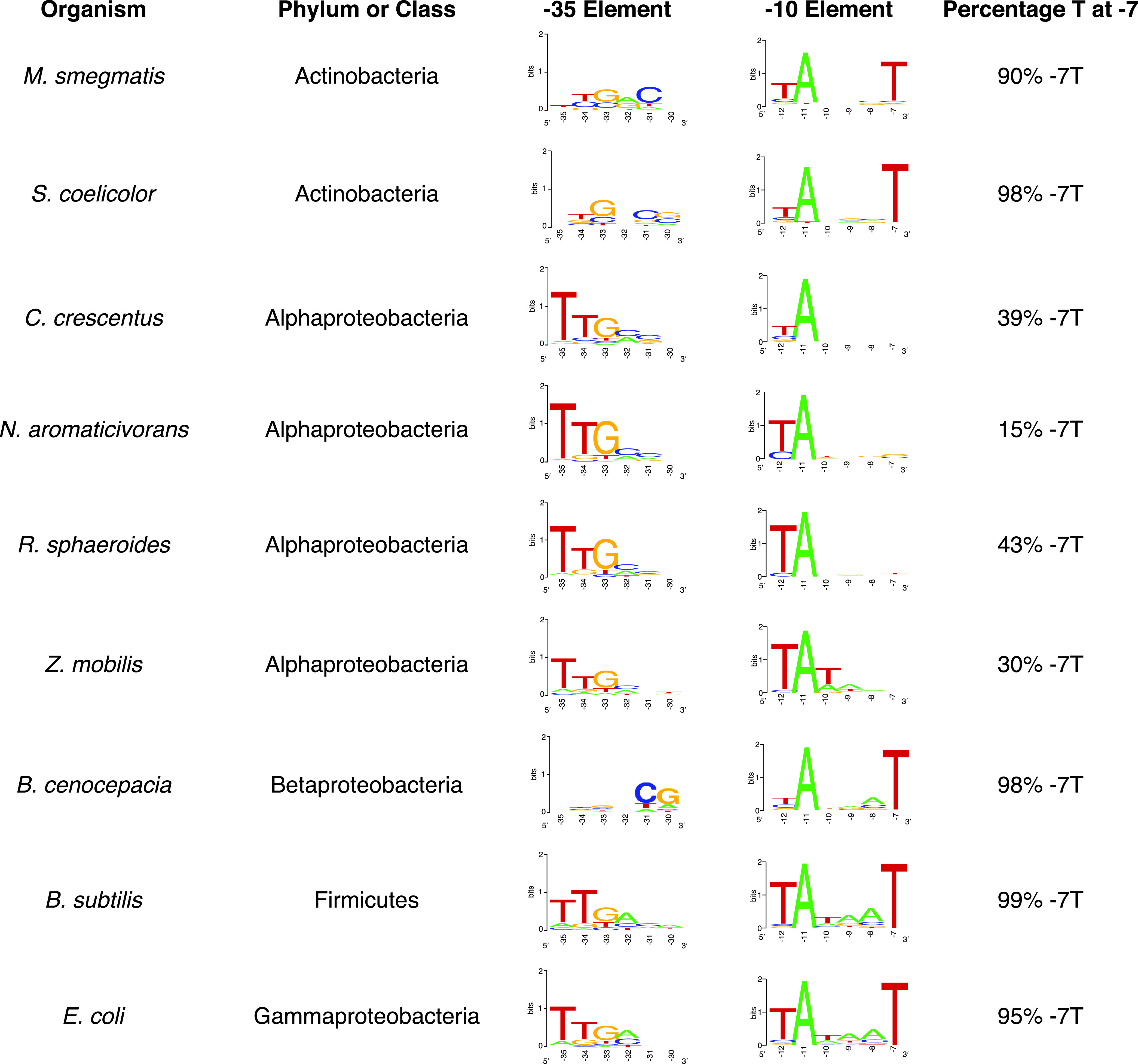
Sequences of MEME-predicted σ^70^-dependent −35 and −10 promoter elements for individual bacterial species. Indicated are the organism’s name, the taxonomic group it belongs to, and the most likely sequences of −35 and −10 elements predicted by the MEME motif finder upstream of the published TSS-seq data for this organism. The last column on the right indicates the percentage of the predicted σ^70^-dependent −10 promoter elements that contain a thymine at position −7 (−7T) relative to the published TSS.

Since σ^70^ homologs are needed for transcription of most genes (25, 27), the majority of the TSSs in these genome-scale data sets are predicted to be derived from σ^70^-dependent promoters. Thus, we hypothesized that including all identified TSSs in each bacterium in our analysis would allow the discovery of overrepresented DNA sequences that corresponded to the σ^70^ promoter sequence. Further, the large number of TSSs analyzed predicts that any activity of alternative σ factors on genome-wide transcription would be minimal. Additionally, most of the TSS data sets were generated under environmental conditions where the activity of many alternative σ factors was predicted to be low, thereby limiting the influence of other promoter motifs on our analysis (Table 1). Using MEME as a motif discovery tool (45, 46), we were able to identify upstream motifs with DNA sequence similarity to −35 and −10 promoter elements that would be predicted to be recognized by the respective housekeeping σ factor based on what is known about σ^70^-promoter interactions in other well-studied bacterial species (Fig. 1; Table S1). Upon examining the overrepresented sequences, we found that motifs identified by MEME are generally conserved across this diverse set of bacterial species and have significant sequence identity to −35 and −10 promoter elements that are known to represent binding sites for σ^70^-containing RNAP in well-studied organisms.

10.1128/mSystems.00526-21.1 TABLE S1 Summary of MEME-identified predicted σ^70^-dependent promoters upstream of TSSs. Download Table S1, XLSX file, 0.7 MB. Copyright © 2021 Myers et al. 2021 Myers et al. https://creativecommons.org/licenses/by/4.0/ This content is distributed under the terms of the Creative Commons Attribution 4.0 International license.

However, the motifs identified by MEME also make predictions about some base-specific differences in promoter elements that are recognized by σ^70^ RNAP holoenzyme across the bacterial phylogeny. For example, TSS-seq data sets show that over 60% of the −10 elements of the *Alphaproteobacteria* R. sphaeroides, Zymomonas mobilis (37, 96), and Caulobacter crescentus (37, 43, 96) lack the thymine at position −7 that is found in over 95% of the E. coli and Bacillus subtilis σ^70^-dependent promoters (Fig. 1). In an extreme case, the MEME motif finder indicates that >80% of the σ^70^-dependent promoters in Novosphingobium aromaticivorans, another member of the *Alphaproteobacteria*, lack a conserved thymine at position −7 (Fig. 1). The low frequency of a thymine at position −7 in *Alphaproteobacteria* σ^70^-dependent promoters contrasts with the prediction made by the MEME motif finders that >90% of the analogous transcription units contain a thymine at this position in *Actinobacteria*, *Betaproteobacteria*, *Firmicutes*, and *Gammaproteobacteria* for which genome-scale TSS data sets are publicly available (Fig. 1).

The MEME motif finder also identified the overrepresented TTG DNA sequences within potential −35 elements that were well conserved across most of the same bacterial species (Fig. 1; Table S1). However, this DNA sequence was not found to be overrepresented in *Betaproteobacteria* and *Actinobacteria* (Fig. 1).

To analyze additional features of these putative σ^70^-dependent promoters across the bacterial phylogeny, we also used the predictions of the MEME motif finder to determine the distance between the −10 and −35 elements and calculate the number of bases between the downstream end of the −10 element and the experimentally determined TSS. This analysis resulted in the same most frequent distance between −35 and −10 elements of the σ^70^-dependent promoters (17 bp) and between the TSS and the −10 element (6 bp) across the species for which genome-scale TSS data are available, suggesting that these features of promoters are conserved across the bacterial phylogeny (Table S1).

**FIG 2:**
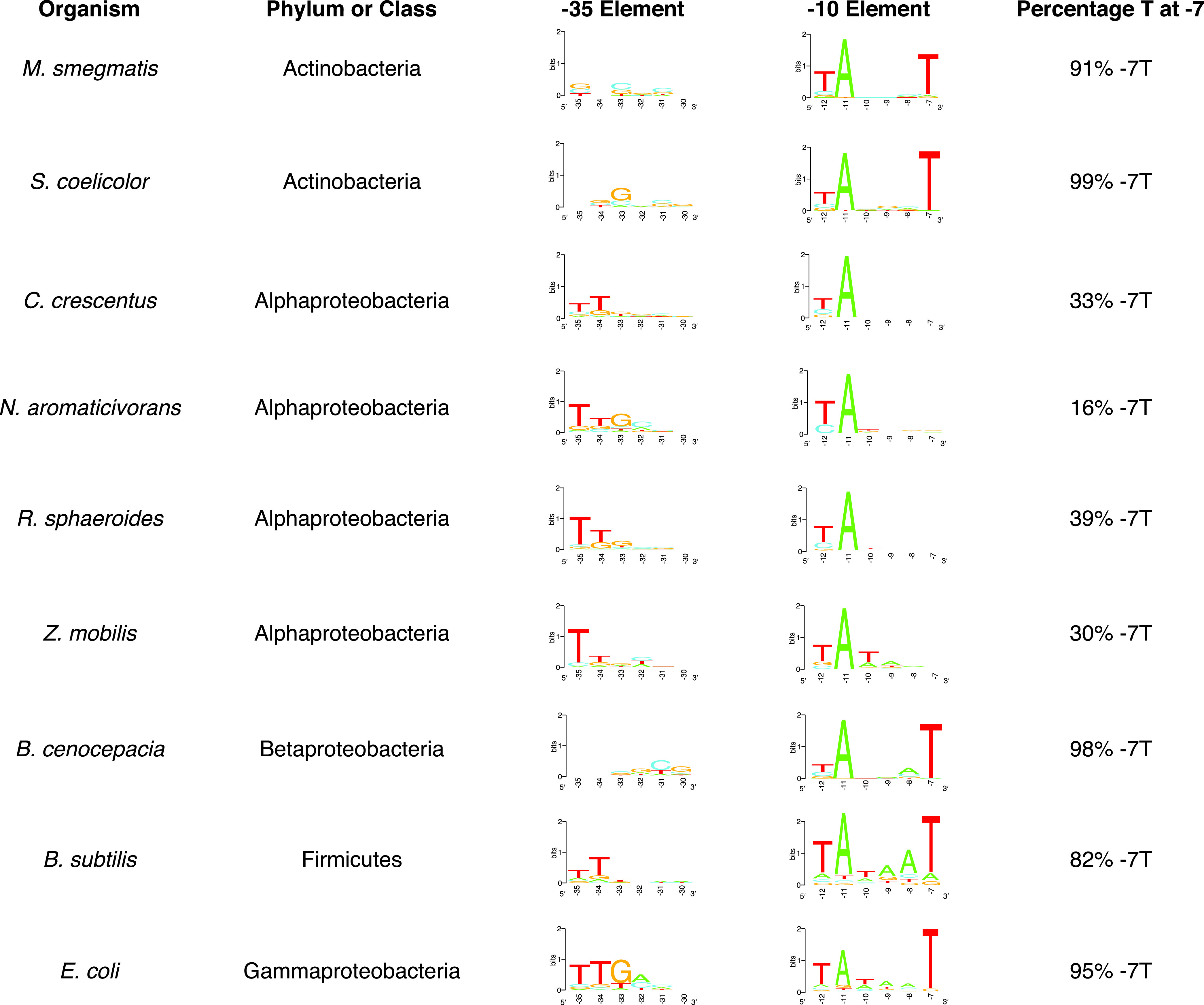
Sequences of Delila-PY-predicted σ^70^-dependent −35 and −10 promoter elements for individual bacterial species. Indicated are the organism’s name, the taxonomic group it belongs to, and the most likely sequences of −35 and −10 elements predicted by Delila-PY upstream of the published TSS-seq data for this organism. The last column on the right indicates the percentage of the predicted σ^70^-dependent −10 promoter elements that contain a thymine at position −7 (−7T) relative to the published TSS.

**FIG 3:**
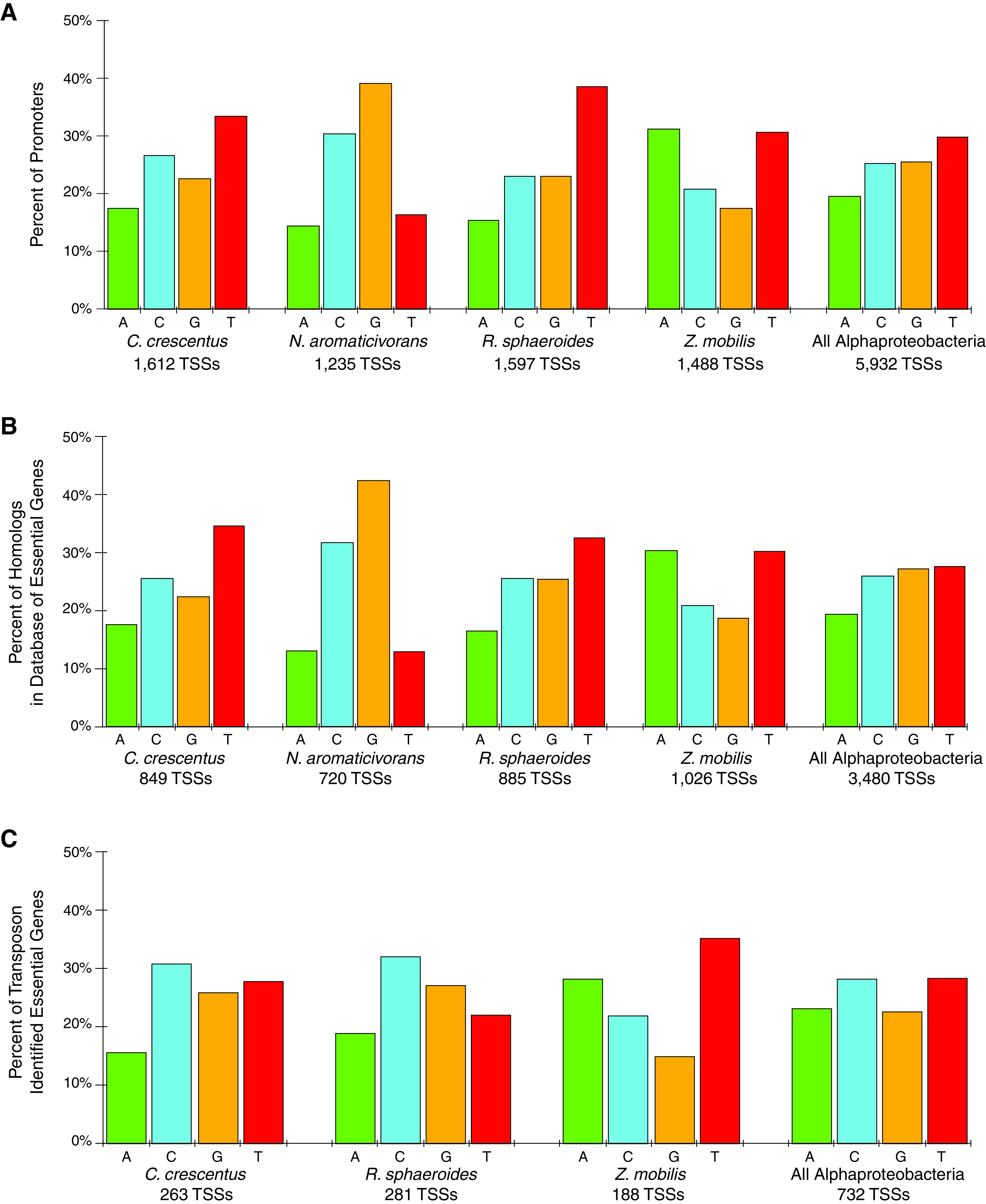
Distribution of −7A (green), −7C (blue), −7G (yellow), and −7T (red) bases within −10 elements upstream of all TSSs as identified by Delila-PY (A), upstream of genes with at least one homolog in the Database of Essential Genes (DEG) (B), and upstream of genes identified as essential using transposon insertion data sets (C) for the bacterial species indicated. The average distribution across all *Alphaproteobacteria* is indicated as “All Alphaproteobacteria.” The number of TSSs identified for each data set is listed below each group of bars.

To test if the prediction of sequences of these promoter elements was influenced by the motif-finding algorithm, we analyzed the same genome-wide TSS-seq data sets using Delila-PY (47), a Python-based pipeline interface with the Delila software suite (48) that uses a different motif-finding method than MEME. The use of Delila-PY to identify DNA sequence motifs upstream of the experimentally mapped TSSs predicted that >60% of the −10 elements of *Alphaproteobacteria* σ^70^-dependent promoters lacked a T at position −7 in all four species examined (Fig. 2; Table S2). This analysis also revealed that, while there are some species-specific differences in the base distribution at position −7, when averaged across all *Alphaproteobacteria*, there is a roughly equal percentage of each base at position −7 (Fig. 3A). In agreement with MEME, Delila-PY predicted that >80% of the non-*Alphaproteobacteria* −10 elements for σ^70^-dependent promoters contained a thymine at position −7 (Fig. 2; Table S2). Further, Delila-PY predicted a lack of conservation for the −35 element for Mycobacterium smegmatis, Streptomyces coelicolor, and Burkholderia cenocepacia (Fig. 2). In total, there was a 60% to 95% agreement between the predicted σ^70^ −35 and −10 promoter elements identified by both the MEME motif finder and Delila-PY across the data sets we analyzed. The fact that similar predictions about the DNA sequences of σ^70^-dependent promoters are made when using either MEME or Delila-PY suggest that the observed differences were not due to a specific algorithm but are likely to be biologically relevant.

10.1128/mSystems.00526-21.2 TABLE S2 Summary of Delila-PY-identified predicted σ^70^-dependent promoters upstream of TSSs. Download Table S2, XLSX file, 1.1 MB. Copyright © 2021 Myers et al. 2021 Myers et al. https://creativecommons.org/licenses/by/4.0/ This content is distributed under the terms of the Creative Commons Attribution 4.0 International license.

In sum, this analysis illustrates that new insights can be obtained from a comparative analysis of genome-scale TSS-seq data across bacterial species. Specifically, it makes a prediction that the −35 and −10 elements of σ^70^-dependent promoters vary significantly across the bacterial phylogeny. In addition, since the MEME motif finder and Delila-PY identified similar overrepresented sequences proximal to the TSS, we conclude that the nucleotide present at position −7 relative to the TSS of σ^70^-dependent promoters is a previously unrecognized feature across many *Alphaproteobacteria*. Below, we focus on predictions about the nature and consequences of the difference in the base at position −7 of the −10 element for predicted σ^70^-dependent promoters within *Alphaproteobacteria*.

### Functional groups associated with the products of genes that are downstream of −7T σ^70^-dependent promoters among *Alphaproteobacteria*.

Because a thymine at position −7 (−7T) relative to the TSS of σ^70^-dependent promoters is present in only a minority of the predicted promoters in *Alphaproteobacteria* (Fig. 1 and 2), we investigated if this small set of transcription units is enriched for gene products that have specific functions across these bacteria. When we analyzed the genes downstream of −7T σ^70^-dependent promoters in *Alphaproteobacteria*, we found that <20% of these encoded homologs of proteins contained in the bacterial Database of Essential Genes (49, 50) (Fig. 3B; Table S2). There was also a roughly equal distribution of all 4 bases at position −7 within promoters upstream of σ^70^-dependent transcription units that encode homologs of these essential proteins (Fig. 3B). We also analyzed the predicted −7T σ^70^-dependent promoters upstream of R. sphaeroides and C. crescentus genes that have been identified as essential in transposon insertion sequencing (Tn-seq) mutant libraries (51, 52) and genes identified as essential in Z. mobilis via microarrays (53). This analysis predicts that <15% of the genes containing −7T σ^70^-dependent promoters in these three species are essential and found that there was no statistical enrichment in these gene products (hypergeometric test, *P* ≤ 0.05) (Fig. 3C; Table S2). Indeed, there is no significant enrichment for any base at position −7 in the σ^70^-dependent promoters that are found upstream of these essential genes (Fig. 3C). Taken together, these data suggest that the genomes of *Alphaproteobacteria* have no significant enrichment for known essential genes downstream of −7T σ^70^-dependent promoters.

**FIG 4:**
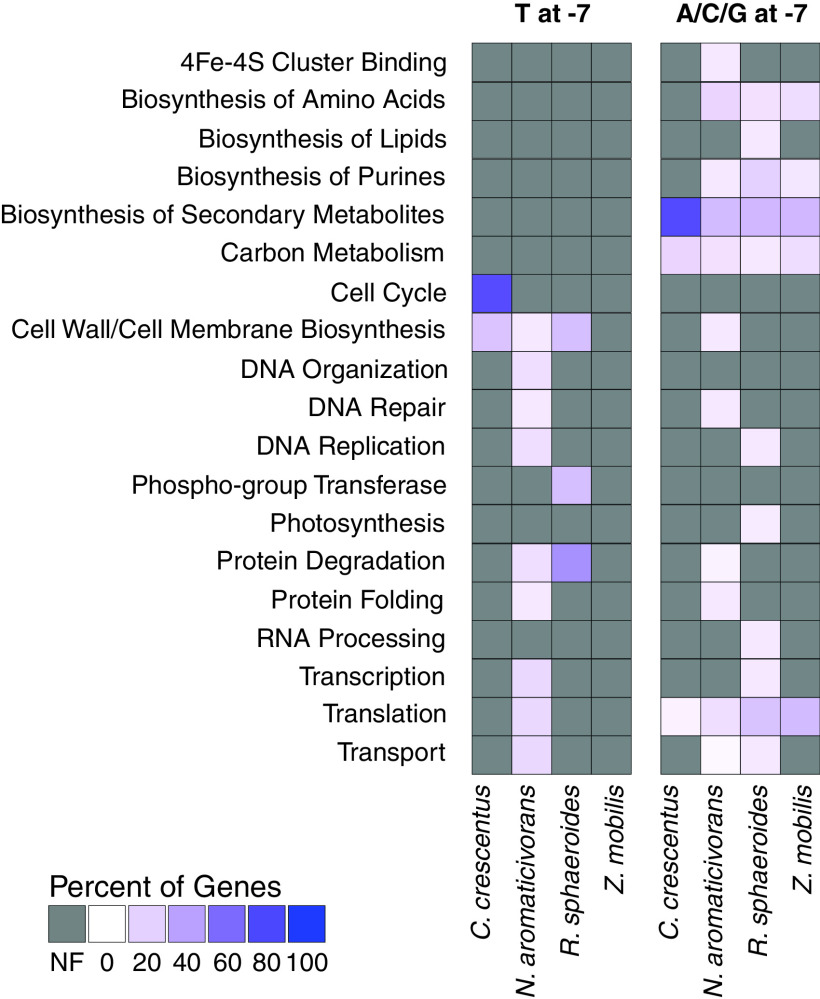
Functional enrichment of genes downstream of predicted −7T σ^70^-dependent promoters (left) or −7A/C/G σ^70^-dependent promoters (right). Colors indicate percentage of all enriched genes within each species present in each cluster (rows) in each organism (columns) using the code shown at the lower left. Darker purple indicates that more enriched genes were present within that individual category. Gray boxes indicate gene sets which show no functional enrichment (NF) for a specific group in the indicated bacterial species.

We also tested for functional enrichment of the products transcribed from genes downstream of predicted *Alphaproteobacteria* −7T σ^70^-dependent promoters. To do this, we analyzed the predicted cellular role of gene products transcribed from *Alphaproteobacteria* transcription units with a −7T σ^70^-dependent promoter with functional groups compiled from the KEGG Brite ontology, the KEGG pathway lists, and GO terms from each organism (54, 55) using a hypergeometric test (an adjusted *P* value of ≤0.1 indicates significant enrichment) (Fig. 4; Table 2; Table S3). This analysis revealed that the annotated functions of gene products downstream of predicted −7T σ^70^-dependent promoters were highly variable among the *Alphaproteobacteria* species for which genome-scale TSS data were available. Indeed, the only functions enriched in more than one *Alphaproteobacteria* were annotated as having roles in cell envelope function and protein degradation (Fig. 4; Table 2; Table S3). Moreover, this analysis revealed that Z. mobilis had no statistically significant enrichment for any functional groups for products encoded by genes downstream of predicted −7T σ^70^-dependent promoters, perhaps reflecting the low number of genes that fall in this category in this bacterium.

**TABLE 2:** Functional enrichment of genes downstream of predicted −7T σ^70^-dependent promoters

Functional group	Species	Subgroup	No. of genes
Cell Cycle	C. crescentus	Cell Cycle (KEGG Brite ccs04112)	12
Cell Wall / Cell Membrane	C. crescentus	Peptidoglycan Metabolic Process (GO:0000270)	4
	*N. aromaticivorans*	Lipid Biosynthesis Proteins (KEGG Brite nar01004)	2
	R. sphaeroides	Peptidoglycan Biosynthetic Process (GO:0009252)	10
DNA Organization	*N. aromaticivorans*	Chromosome and Associated Proteins (KEGG Brite nar03036)	4
DNA Repair	*N. aromaticivorans*	DNA Repair and Recombination Proteins (KEGG Brite nar03400)	5
DNA Replication	*N. aromaticivorans*	DNA Replication Proteins (KEGG Brite nar03032)	3
Phospho-Group Transferase	R. sphaeroides	Transferase Activity Transferring Phosphorus-Containing Groups (GO:0016772)	10
Protein Degradation	*N. aromaticivorans*	Peptidases and Inhibitors (KEGG Brite nar01002)	4
		Protein Catabolic Process (GO:0030163)	3
	R. sphaeroides	Peptidases and Inhibitors (KEGG Brite rsp01002)	14
		Protein Catabolic Process (GO:0030163)	4
Protein Folding	*N. aromaticivorans*	Chaperones and Folding Catalysts (KEGG Brite nar03110)	5
Transcription	*N. aromaticivorans*	Transcription Factors (KEGG Brite nar03000)	5
		Two-Component System (KEGG Brite nar02022)	2
		Transcription Machinery (KEGG Brite nar03021)	2
Translation	*N. aromaticivorans*	Mitochondrial Biogenesis (KEGG Brite nar03029)	4
		Exosome (KEGG Brite nar04147)	3
		Ribosome Biogenesis (KEGG Brite nar03009)	3
Transport	*N. aromaticivorans*	Transporters (KEGG Brite nar02000)	8

10.1128/mSystems.00526-21.3 TABLE S3 Functional group enrichment for products of genes downstream of predicted −7T σ^70^-dependent promoters and −7A/C/G σ^70^-dependent promoters. Download Table S3, XLSX file, 0.07 MB. Copyright © 2021 Myers et al. 2021 Myers et al. https://creativecommons.org/licenses/by/4.0/ This content is distributed under the terms of the Creative Commons Attribution 4.0 International license.

**FIG 5:**
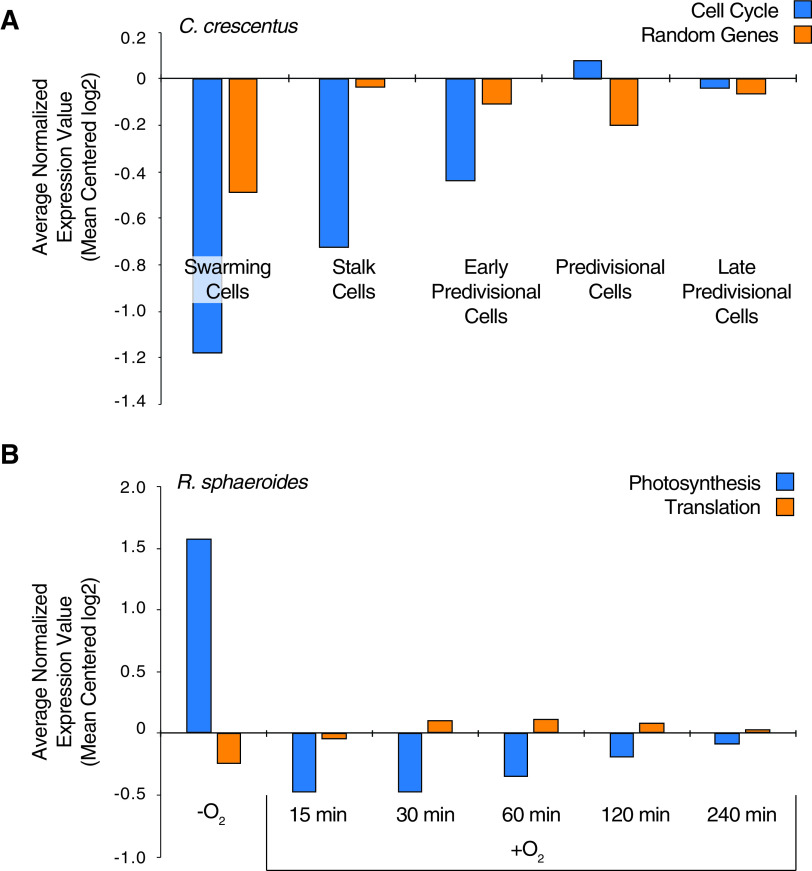
Average transcript abundance values for selected functional groups in C. crescentus (A) and R. sphaeroides (B). (A) Average transcript abundance values for genes downstream of predicted −7T σ^70^-dependent promoters involved in the cell cycle (blue) and the same number of randomly selected genes downstream of predicted −7T σ^70^-dependent promoters (orange) in C. crescentus during the cell cycle. (B) Average transcript abundance values for genes downstream of predicted −7A/C/G σ^70^-dependent promoters involved in photosynthesis (blue) and translation (orange) in R. sphaeroides as a function of time after exposing an anaerobic culture to oxygen.

In the three *Alphaproteobacteria* for which genome-wide TSS data are available, we found evidence for functional enrichments unique to the individual organisms. For example, in *N. aromaticivorans*, the largest number of enriched functional groups of proteins encoded by genes downstream of −7T σ^70^-dependent promoters were predicted to function in translation, transport, transcription, protein folding, DNA organization, DNA repair, and DNA replication (Fig. 4; Table 2; Table S3), as well as some proteins being predicted to allow *N. aromaticivorans* to metabolize aromatic compounds (56–60). In R. sphaeroides, the products encoded by genes downstream of predicted −7T σ^70^-dependent promoters were enriched for phosphor-group transfer, including sensor histidine kinases of two-component regulatory systems (Fig. 4; Table 2; Table S3). In C. crescentus, the products of genes downstream of predicted −7T σ^70^-dependent promoters were enriched in those involved in the cell cycle changes of this alphaproteobacterium (Fig. 4; Table 2; Table S3) (61). Because of this predicted enrichment, we asked if the genes in C. crescentus downstream of a predicted −7T σ^70^-dependent promoter exhibited any cell cycle changes in transcription in published RNA-seq data set (62). This analysis revealed an increase of the average abundance of transcripts derived from the cell cycle genes downstream of predicted −7T σ^70^-dependent promoters over the cell cycle (Fig. 5A). We did not find a similar pattern in cell cycle-specific transcript abundance when we analyzed the same number of randomized genes downstream of predicted −7T σ^70^-dependent promoters for genes that encode proteins with different functions (Fig. 5A). Taken together, these results indicate that genes downstream of predicted −7T σ^70^-dependent promoters do not encode common cellular functions across the *Alphaproteobacteria*. Instead, they predict that −7T σ^70^-dependent promoters are found upstream of transcription units that encode proteins responsible for diverse and possibly lifestyle-specific sets of cellular functions.

### *Alphaproteobacteria* proteins transcribed from genes downstream of σ^70^-dependent −7A/C/G promoters perform essential and core cellular functions.

We also examined the predicted functions of *Alphaproteobacteria* proteins transcribed from genes downstream of predicted σ^70^-dependent promoters containing an adenine, cytosine, or guanine at position −7 (−7A/C/G promoters). In this case, we found that there was a high percentage of homologous proteins in the bacterial Database of Essential Genes (DEG) (49, 50) that were encoded by transcription units that contained predicted −7A/C/G σ^70^-dependent promoters (Fig. 3B; Table S2) but no significant difference in distribution of bases at this position within these promoters. Further, we found statistically significant enrichment of transposon-identified essential genes located downstream of predicted −7A/C/G σ^70^-dependent promoters (51–53) (Fig. 3C; Table S2) (hypergeometric test, *P* ≤ 0.05). Examining the individual base distributions at position −7 again showed no significant enrichment (Fig. 3C). These analyses suggest that *Alphaproteobacteria* have a higher percentage of −7A/C/G σ^70^-dependent promoters upstream of genes encoding known essential functions than those containing a thymine at this position and that an A, C, and G at position −7 are roughly equally distributed in all the *Alphaproteobacteria* included in our analysis (Fig. 3).

We also tested for functional enrichments in the proteins transcribed from genes that are downstream of predicted −7A/C/G σ^70^-dependent promoters. This analysis identified several functional groups enriched in multiple *Alphaproteobacteria* species for products encoded by genes that are downstream of predicted −7A/C/G σ^70^-dependent promoters (Fig. 4; Table 3; Table S3). The enriched functional groups shared among all 4 *Alphaproteobacteria* species were gene products involved in translation, central carbon metabolism, and the biosynthesis of secondary metabolites (Fig. 4; Table 3; Table S3). The biosynthesis of purines (three species), amino acid biosynthesis gene products (three species), and transporters (two species) was enriched in the products of genes downstream of predicted −7A/C/G σ^70^-dependent promoters in a subset of the *Alphaproteobacteria* for which genome-wide TSS data sets were available (Fig. 4; Table 3; Table S3).

**TABLE 3:** Functional enrichment of genes downstream of predicted −7A/C/G σ^70^-dependent promoters

Functional group	Species	Subgroup	No. of genes
4Fe-4S Cluster Binding	*N. aromaticivorans*	4Fe-4S Cluster Binding (GO:0051539)	20
Biosynthesis of Amino Acids	*N. aromaticivorans*	Biosynthesis of Amino Acids (KEGG Pathways nar01230)	64
		Cellular Amino Acid Biosynthetic Process (GO:0008652)	38
		Valine, Leucine, and Isoleucine Biosynthesis (KEGG Pathways nar00290)	10
		Branched-Chain Amino Acid Biosynthetic Process (GO:0009082)	9
		C5-Branched Dibasic Acid Metabolism (KEGG Pathways nar00660)	9
		Cysteine and Methionine Metabolism (KEGG Pathways nar00270)	17
		Lysine Biosynthetic Process via Diaminopimelate (GO:0009089)	7
		Lysine Biosynthetic Process (GO:0009085)	7
		Leucine Biosynthetic Process (GO:0009098)	5
		Isoleucine Biosynthetic Process (GO:0009097)	6
		Lysine Biosynthesis (KEGG Pathways nar00300)	9
		Isoprenoid Biosynthetic Process (GO:0008299)	6
	R. sphaeroides	Biosynthesis of Amino Acids (KEGG Pathways rsp01230)	50
		Cellular Amino Acid Biosynthetic Process (GO:0008652)	29
		Histidine Biosynthetic Process (GO:0000105)	7
		Threonine Biosynthetic Process (GO:0009088)	4
		Lysine Biosynthetic Process (GO:0009085)	6
		Lysine Biosynthetic Process via Diaminopimelate (GO:0009089)	6
		Lysine Biosynthesis (KEGG Pathways rsp00300)	9
		Cysteine and Methionine Metabolism (KEGG Pathways rsp00270)	15
		Methionine Biosynthetic Process (GO:0009086)	7
	Z. mobilis	Biosynthesis of Amino Acids (KEGG Pathways zmo01230)	29
Biosynthesis of Lipids	R. sphaeroides	DNA Replication (KEGG Pathways rsp03030)	8
Biosynthesis of Purines	*N. aromaticivorans*	Purine Nucleotide Biosynthetic Process (GO:0006164)	11
		Purine Metabolism (KEGG Pathways nar00230)	18
		‘De Novo’ IMP Biosynthetic Process (GO:0006189)	8
	R. sphaeroides	Purine Nucleotide Biosynthetic Process (GO:0006164)	10
	Z. mobilis	Exosome (KEGG Brite zmo04147)	20
Biosynthesis of Secondary Metabolites	C. crescentus	Biosynthesis of Secondary Metabolites (KEGG Pathways ccs01110)	26
		Biosynthesis of Cofactors (KEGG Pathways ccs01240)	7
	*N. aromaticivorans*	Biosynthesis of Secondary Metabolites (KEGG Pathways nar01110)	141
		Heme Biosynthetic Process (GO:0006783)	5
	R. sphaeroides	Biosynthesis of Secondary Metabolites (KEGG Pathways rsp01110)	119
		Biosynthesis of Cofactors (KEGG Pathways rsp01240)	61
	Z. mobilis	Biosynthesis of Secondary Metabolites (KEGG Pathways zmo01110)	58
Biosynthesis of Purines	*N. aromaticivorans*	Purine Nucleotide Biosynthetic Process (GO:0006164)	11
		Purine Metabolism (KEGG Pathways nar00230)	18
		‘De Novo’ IMP Biosynthetic Process (GO:0006189)	8
	R. sphaeroides	Purine Nucleotide Biosynthetic Process (GO:0006164)	10
	Z. mobilis	Exosome (KEGG Brite zmo04147)	20
Carbon Metabolism	C. crescentus	One-Carbon Metabolic Process (GO:0006730)	7
	*N. aromaticivorans*	2-Oxocarboxylic Acid Metabolism (KEGG Pathways nar01210)	16
		Carbon Metabolism (KEGG Pathways nar01200)	47
		Glycolytic Process (GO:0006096)	7
		Citrate Cycle (TCA Cycle (KEGG Pathways nar00020)	14
		Gluconeogenesis (GO:0006094)	6
		TCA Cycle (GO:0006099)	9
		Pentose Phosphate Pathway (KEGG Pathways nar00030)	10
	R. sphaeroides	Carbon Metabolism (KEGG Pathways rps01200)	43
		2-Oxocarboxylic Acid Metabolism (KEGG Pathways rsp01210)	12
		TCA Cycle (GO:0006099)	8
	Z. mobilis	Carbon Metabolism (KEGG Pathways zmo01200)	18
		Glycolysis/Gluconeogenesis (KEGG Pathways – zmo00010)	11
		Pentose Phosphate Pathway (KEGG Pathways zmo00030)	5
		Carbohydrate Metabolic Process (GO:0005975)	10
Cell Wall / Membrane	*N. aromaticivorans*	Polysaccharide Biosynthetic Process (GO:0000271)	7
		Phospholipid Biosynthetic Process (GO:0008654)	7
DNA Repair	*N. aromaticivorans*	DNA Repair (GO:0006281)	19
		Cellular Response to DNA Damage Stimulus (GO:0006974)	15
		DNA Topoisomerase Type II (Double Strand Cut) (GO:0003918)	4
		Base Excision Repair (KEGG Pathways nar03410)	8
DNA Replication	R. sphaeroides	DNA Replication (KEGG Pathways rsp03030)	8
Photosynthesis	R. sphaeroides	Porphyrin-Containing Compound Biosynthetic Process (GO:0006779)	8
		Porphyrin and Chlorophyll Metabolism (KEGG Pathways rsp00860)	22
		Protoporphyrinogen IX Biosynthetic Process (GO:0006782)	7
		Chlorophyll Biosynthetic Process (GO:0015995)	12
		Coproporphyrinogen Oxidase Activity (GO:0004109)	4
		Carbon Fixation in Photosynthetic Organisms (KEGG Pathways rsp00710)	11
Protein Degradation	*N. aromaticivorans*	Proteolysis (GO:0006508)	28
		Serine-Type Endopeptidase Activity (GO:0004252)	10
Protein Folding	*N. aromaticivorans*	Peptidyl-Prolyl Cis-Trans Isomerase Activity (GO:0003755)	7
		Protein Peptidyl-Prolyl Isomerization (GO:0000413)	7
		Protein Folding (GO:0006457)	8
RNA Processing	R. sphaeroides	RNA Phosphodiester Bond Hydrolysis Exonucleolytic (GO:0090503)	4
Transcription	R. sphaeroides	Transcription Machinery (KEGG Brite rsp03021)	12
Translation	C. crescentus	Translation (GO:0006412)	3
	*N. aromaticivorans*	Translation (GO:0006412)	50
		Aminoacyl-tRNA Ligase Activity (GO:0004812)	19
		tRNA Aminoacylation for Protein Translation (GO:0006418)	14
		Aminoacyl-tRNA Biosynthesis (KEGG Pathways nar00970)	19
		tRNA Processing (GO:0008033)	14
		tRNA Binding (GO:0000049)	12
		tRNA Aminoacylation (GO:0043039)	5
	R. sphaeroides	Translation (GO:0006412)	54
		Aminoacyl-tRNA Biosynthesis (KEGG Pathways rsp00970)	44
		Aminoacyl-tRNA Ligase Activity (GO:0004812)	18
		Transfer RNA Biogenesis (KEGG Brite rsp03016)	33
		tRNA Aminoacylation for Protein Translation (GO:0006418)	14
		Translation Factors (KEGG Brite rsp03012)	13
		Mitochondrial Biogenesis (KEGG Brite rsp03029)	20
		tRNA Binding (GO:0000049)	15
		RNA Binding (GO:0003723)	31
		Non-Coding RNAs (KEGG Brite rsp03100)	25
		Translational Termination (GO:0006415)	5
		Ribosome (GO:0005840)	21
		tRNA Processing (GO:0008033)	12
		Structural Constituent of Ribosome (GO:0003735)	20
		Ribosome Biogenesis (KEGG Brite rsp03009)	17
		Translation Elongation Factor Activity (GO:0003746)	6
		Translation Elongation (GO:0006414)	6
	Z. mobilis	Translation (GO:0006412)	29
		Ribosome (KEGG Brite zmo03011)	38
		Ribosome (KEGG Brite zmo03010)	19
		Structural Component of Ribosome (GO:0003735)	19
		Ribosome (GO:0005840)	20
Transport	*N. aromaticivorans*	Protein Export (KEGG Pathways nar03060)	10
	R. sphaeroides	Protein Export (KEGG Pathways rsp03060)	11
		Bacterial Secretion System (KEGG Pathways rsp03070)	10

In addition, this analysis showed enrichment of several other groups of gene products that are transcribed from predicted −7A/C/G σ^70^-dependent promoters in single bacterial species (Fig. 4; Table 3; Table S3). One example of this is the enrichment of genes whose products are involved in photosynthesis in R. sphaeroides, the only phototrophic alphaproteobacterium for which genome-wide TSS data sets are available (Fig. 4; Table 3; Table S3). Consistent with the bioinformatically predicted function of the −7A/C/G σ^70^-dependent promoters that are upstream of genes encoding proteins involved in photosynthesis, there is a significant reduction in abundance of transcripts encoding proteins involved in photosynthesis and only a slight increase in abundance of those encoding translation functions after photosynthetic cells are shifted to nonphotosynthetic conditions (Fig. 5B) (63). We also found that products encoded by genes transcribed from predicted −7A/C/G σ^70^-dependent promoters that function in lipid biosynthesis and transcription were among those enriched only in R. sphaeroides. The genes transcribed from predicted −7A/C/G σ^70^-dependent promoters whose products are involved in lipid biosynthesis may play a role in forming the membrane invaginations that house the photosynthetic apparatus of this organism (64), while the genes encoding alternative σ factors in this group (*rpoE*, *rpoH1*, and *rpoH2*) may play a role in the R. sphaeroides response to singlet oxygen and heat or envelope stress (Fig. 4; Table 3; Table S3) (65–70).

In another example, predicted −7A/C/G σ^70^-dependent promoters in *N. aromaticivorans* were overrepresented upstream of transcription units that encode iron-sulfur proteins, enzymes in cell wall/cell membrane biosynthesis, DNA repair, protein degradation, and protein folding (Fig. 4; Table 3; Table S3). Phenolic compounds metabolized by *N. aromaticivorans* are known to damage bacterial cell membranes and other macromolecules, suggesting that this gene is associated with a lifestyle of this alphaproteobacterium (71, 72). Further, several of the iron-sulfur proteins transcribed from genes downstream of predicted −7A/C/G σ^70^-dependent promoters function in the tricarboxylic acid (TCA) cycle, which assimilates the products of aromatic metabolism into *N. aromaticivorans* cellular biomass (58, 73). Below, we discuss the importance of finding that a majority of *Alphaproteobacteria* genes that are transcribed from predicted −7A/C/G σ^70^-dependent promoters include those involved in many cellular functions.

## DISCUSSION

The initiation of transcription requires the recognition and binding of RNAP to specific promoter DNA sequences, an event that requires a σ factor, and can depend on other proteins and small molecule ligands (3, 4). A variety of studies have helped predict the promoter sequence in some well-studied bacterial species, but access to genome-scale maps of TSSs at the nucleotide level provides an opportunity to catalog and compare promoter sequences across the bacterial phylogeny. Here, we predicted promoter sequences using bioinformatic analysis of published genome-scale TSS-seq data sets. Using the MEME and Delila-PY motif-finding algorithms (45–47), we predicted differences in the −35 (*Actinobacteria* and *Betaproteobacteria*) and −10 (*Alphaproteobacteria*) promoter elements that are recognized by the housekeeping σ factor (σ^70^). Below, we discuss the biochemical and functional consequences of the differences in predicted σ^70^-dependent promoters across these taxa.

### Features of σ^70^-dependent promoter recognition that are conserved across phyla.

**FIG 6:**
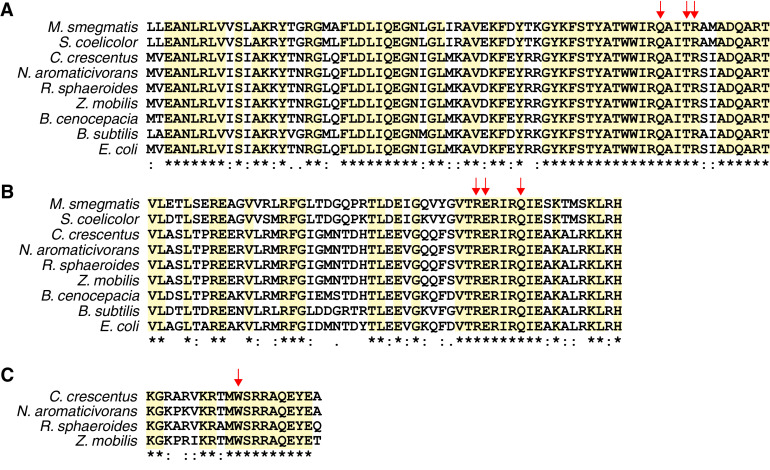
Amino acid alignment of region 2 (A) and region 4 (B) of the housekeeping σ factor and portion of CarD homologs (C) from the indicated bacterial species. Asterisks and highlighting indicate fully conserved residues, colons indicate conservation between residues with strongly similar properties, and periods indicate conservation between residues with weakly similar properties (94, 95). Conserved residues involved in predicted σ^70^-dependent −10 promoter binding (A), −35 promoter binding (B), or key functional residue in CarD (C) are indicated by red arrows.

The −10 and −35 elements of bacterial promoters make specific contacts with separate regions of σ factors. From mechanistic studies, amino acids in σ^70^ region 2.4 make specific contacts with the −10 element in cognate promoters (27, 74). Comparison of the sequence of σ^70^ region 2 in the phyla we examined shows a high level of amino acid conservation, including Q437, T440, and R441 (using residue numbers of E. coli σ^70^) (Fig. 6A), which recognize the −10 region of the promoter (10). These residues are also conserved in the *Alphaproteobacteria*, where the sequences of the −10 elements in the majority of the predicted σ^70^ promoters lack a thymine at position −7 that is highly conserved in many other promoters that are recognized by this σ factor. Similarly, the −35 promoter elements interact specifically with σ^70^ region 4 (10). Comparison of σ^70^ region 4 among the bacteria studied here also shows a high degree of amino acid conservation, including residues that recognize the −35 sequence: R584, E585, and Q589 (using residues numbers of E. coli σ^70^) (Fig. 6B). Indeed, these σ^70^ region 4 amino acids are conserved in M. smegmatis, S. coelicolor, and B. cenocepacia, bacteria that lack a −35 TTG sequence that is conserved across the phyla (Fig. 1 and 2). This suggests that few, if any, differences in σ^70^ exist to account for the observed differences in −35 elements of M. smegmatis, S. coelicolor, and B. cenocepacia and the −10 elements of *Alphaproteobacteria*.

### *Alphaproteobacteria* genes for essential and core metabolic functions contain −7A/C/G σ^70^-dependent promoters.

It was previously proposed that there are differences in the −10 promoter elements of *Alphaproteobacteria*, based on examination of a small number of transcription units (75, 76) or organisms (37, 96). In this study, we used published genome-scale TSS data sets to show that −7T is widely conserved across the bacterial phylogeny, except for the *Alphaproteobacteria*.

We also examined the biological implications of this variance in the sequence of the −10 element of σ^70^-dependent promoters in *Alphaproteobacteria*. We found that essential genes were more likely to be transcribed from predicted −7A/C/G σ^70^-dependent promoters in several *Alphaproteobacteria*. This finding was unexpected given the conservation of amino acid residues in σ^70^ region 2 that recognize the −10 element sequence, and it suggests that there are different requirements for transcription initiation in *Alphaproteobacteria* (see below). We also found that the functions of proteins transcribed from genes downstream of predicted −7A/C/G σ^70^-dependent promoters were often shared among the *Alphaproteobacteria*. This so-called core regulon of genes that are downstream of −7A/C/G σ^70^-dependent promoters includes proteins that function in translation, carbon metabolism, and biosynthesis of amino acids, purines, and secondary metabolites. These findings suggest that there has been a reprogramming of promoter architecture within the *Alphaproteobacteria* to place −7A/C/G σ^70^-dependent promoters upstream of both essential genes and ones that encode numerous cellular functions. The presence of a core *Alphaproteobacteria* regulon that contains predicted −7A/C/G σ^70^-dependent promoters makes it tempting to propose that this reprogramming occurred after the *Alphaproteobacteria* diverged. Analysis of TSS data sets from other members of the bacterial phylogeny is needed to test this hypothesis.

However, we also found *Alphaproteobacteria* −7T σ^70^-dependent promoters upstream of genes that encode proteins with critical functions, including cell cycle genes in C. crescentus and cell wall/cell membrane biosynthesis genes in multiple members of this phylum. In addition, some *Alphaproteobacteria* showed enrichment for different bases at position −7 of σ^70^-dependent promoters upstream of genes that were linked to individual lifestyles. For example, R. sphaeroides showed enrichment for predicted −7A/C/G σ^70^-dependent promoters upstream of genes encoding proteins involved in photosynthesis, while C. crescentus showed enrichment for predicted −7T σ^70^-dependent promoters upstream of genes encoding products involved in their cell cycle developmental program. In contrast, *N. aromaticivorans* contained a large number of enriched functional groups transcribed from genes downstream of both −7T and −7A/C/G σ^70^-dependent promoters. If the latter finding reflects the acquisition by *N. aromaticivorans* of transcription units from a variety of bacteria which allow it to metabolize aromatic compounds, then future analysis of other aromatic-metabolizing *Alphaproteobacteria* (77) might shed light on core or extended regulons for this metabolic capacity. We could not identify functional enrichments for Z. mobilis proteins transcribed from genes downstream of either −7T promoters or −7A/C/G σ^70^-dependent promoters, but this might reflect the number of genes annotated with unknown function and the lack of metabolic analyses of this alphaproteobacterium. Further studies of *Alphaproteobacteria* are needed to better understand the roles of proteins encoded by genes downstream of −7T and −7A/C/G σ^70^-dependent promoters in their lifestyles.

### The potential role of CarD at *Alphaproteobacteria* σ^70^-dependent promoters.

In organisms in which the majority of −10 elements contain a −7T, the presence of other bases at this position often reduces their activity, creates a promoter which requires different base patterns to compensate, or requires another protein to stimulate transcription (78–80, 96). The transcription factor CarD may play such a stimulatory role in *Alphaproteobacteria*, since it increased transcription from several R. sphaeroides σ^70^-dependent −7A/C/G promoters *in vitro* (96), possibly by stabilizing open complex formation by RNAP (28, 29). CarD is essential in several *Alphaproteobacteria*, and a residue required for CarD function (W86) (29, 30) (red arrow in Fig. 6C) is conserved across the *Alphaproteobacteria* we studied, suggesting that this protein also activates transcription in these species (Fig. 6C). Together, these data suggest that CarD homologs enhance transcription by σ^70^-containing RNAP, perhaps by compensating for the lack of a thymine at position −7 in the −10 element of *Alphaproteobacteria*.

Lateral gene transfer (LGT) is common within *Alphaproteobacteria* and with other phyla (81, 82), and it is proposed to be a key component of proteobacterial evolution (83). This raises the possibility that LGT of CarD and transcription units containing −7A/C/G σ^70^-dependent promoters played an important role in both the evolution of the *Alphaproteobacteria* and their branching from other taxa. Additional analysis of alphaproteobacterial species could lead to a better understanding of any evolutionary link between CarD and transcription initiation.

### Potential impacts of promoter differences on biotechnology.

The unique features of *Alphaproteobacteria* σ^70^-dependent promoters described here and elsewhere (37, 96) highlight the importance of analyzing multiple phyla to gain a more complete picture of transcription initiation. For example, the paradigms for σ^70^-dependent sequence motifs developed in other bacteria might not accurately predict the presence of alphaproteobacterial promoters. The ability to control activity of alphaproteobacterial promoters has practical applications, since they have biochemical and metabolic pathways that would be beneficial to harness for various biotechnology applications. These include the conversion of lignin-derived and other aromatic compounds into valuable products by *N. aromaticivorans* (56, 58–60) and the ability of R. sphaeroides to harvest solar energy, fix atmospheric nitrogen and CO_2_, and produce hydrogen and other valuable chemicals (84–88). Future efforts to engineer these and other bacteria will be enhanced by a better understanding of promoter architecture and the role of proteins like CarD in transcription initiation.

### Conclusion.

By analyzing published genome-scale TSS data sets from species across the bacterial phylogeny, we found that *Actinobacteria* and *Betaproteobacteria* σ^70^-dependent promoters lack conserved bases in their predicted −35 elements. We further found that the base at the −7 position of the −10 elements in over 60% of the σ^70^-dependent promoters in several diverse *Alphaproteobacteria* differs from that found in many other phyla, and we propose that CarD plays a role in activating transcription from *Alphaproteobacteria* σ^70^-dependent promoters. Our findings highlight the importance of studying numerous bacterial species to increase our understanding of transcription and engineer members of the *Alphaproteobacteria* as well as other bacterial phyla for processes of medical, agricultural, environmental, and biotechnological importance.

## MATERIALS AND METHODS

### Data sets.

We used published TSS data from M. smegmatis and S. coelicolor (*Actinobacteria*) (40, 41), C. crescentus, *N. aromaticivorans*, R. sphaeroides, and Z. mobilis (*Alphaproteobacteria*) (37, 38, 43), B. cenocepacia (*Betaproteobacteria*) (39), B. subtilis (*Betaproteobacteria*) (44), and E. coli (*Gammaproteobacteria*) (42). All identified TSSs were used in our analysis; there was no attempt to remove any TSSs potentially downstream of alternative σ factors. Genome sequence files (e.g., GFF, GenBank, and genome FASTA) for each species were obtained from NCBI (89) using the following accession numbers for each species: M. smegmatis, NC_008596.1; S. coelicolor, NC_003888.3; C. crescentus, NC_011916.1; *N. aromaticivorans*, NC_007794.1; R. sphaeroides, NC_007493.2; Z. mobilis, NZ_CP023715.1; B. cenocepacia, AM747720.1; B. subtilis, NC_000964.3; and E. coli, NC_000913.3.

### Promoter motif prediction.

The MEME motif finder (version 5.1.0) was used to predict σ^70^-dependent promoter elements upstream of each TSS (45, 46). To identify the −10 promoter element, the nucleotide sequence from −19 to −5 relative to each identified TSS was analyzed using the “zoops” method (minimum width of 9 bp, maximum width of 10 bp, no palindromic motifs) in MEME. The motif with the most hits and lowest *P* value was chosen for each species. The predicted −10 elements all had overrepresented TA nucleotides at positions −12 and −11 relative to the TSS. The percentage of thymine at position −7 was calculated relative to the TA dinucleotide within each predicted −10 promoter element. If identical sequences were used from multiple closely spaced TSSs upstream of the same gene, the duplications were not analyzed to determine the position −7 base percentages. To identify DNA sequences of potential −35 elements in each data set, the positions −40 to −28 relative to each identified TSS with an identified −10 element were analyzed using MEME and the settings described above. Motifs for publication were constructed using WebLogo (90). Distances between the −35 and −10 elements and between the −10 element and position +1 were determined using custom Python scripts.

Delila-PY (47) was also used to predict σ^70^-dependent promoter motifs (48, 91). The DNA sequences in predicted −10 elements were identified by searching −15 to −5 relative to each TSS using default parameters for Delila-PY. The motif with the highest information content is, by default, reported by Delila-PY and was used in our analyses. The percentage of a thymine base (T) at position −7 was calculated as described above. To identify the DNA sequences in −35 elements for each species, the positions −37 to −27 relative to each identified TSS with an identified −10 element were analyzed using the default Delila-PY settings, and the predicted logos are reported in our analyses. We used the Delila-PY −10 promoter element predictions for subsequent analysis due to the larger number of predicted σ^70^-dependent promoters generated by this tool.

### Determining gene essentiality.

Predicted σ^70^-dependent promoters identified by Delila-PY were split into two categories based on the identity of the base at position −7: −7T and −7A/C/G. For each group, the genes downstream of each predicted σ^70^ promoter were searched against the Database of Essential Genes (DEG), version 15 (49). The amino acid sequence of each gene downstream of a predicted σ^70^-dependent promoter was searched against the protein sequences of all bacterial essential genes in DEG 15 using BLAST (version 2.9.0) (92) with a E value threshold of 1 × 10^−10^. Genes with at least one match to a protein sequence within the DEG list in any bacterial species were considered essential for this analysis.

The genes within the −7T and the −7A/C/G groups were also compared to essential genes identified by transposon insertion sequencing (Tn-seq) for R. sphaeroides and C. crescentus (51, 52) or transposon insertion identification via microarrays for Z. mobilis (53). Statistical significance of the number of essential genes within each group and species was determined using a hypergeometric test, with an adjusted *P* value of ≤0.05 being considered significant.

### Functional enrichment.

Functions of gene products downstream of predicted σ^70^-dependent promoters in the −7T and −7A/C/G groups were obtained from the NCBI GenBank file for each species. These predicted functions were mapped to organized protein functional groups from the KEGG Brite ontology, KEGG Pathways, and GO terms (54, 55). The comparison was performed using a hypergeometric test with an adjusted *P* value of ≤0.1 as a threshold for significant enrichment. Subgroups were combined into supergroups by binning similar cellular functions.

### Protein sequence alignments.

Protein sequences of RNA polymerase σ^70^ and CarD homologs were obtained from the GenBank files mentioned above. Clustal Omega (93–95) was used to align the sequences using default parameters.
